# Cultural patterns and outcome of umbilical cord care among caregivers in Africa: a systematic review

**DOI:** 10.1097/MS9.0000000000000762

**Published:** 2023-06-14

**Authors:** Monica Agianipe Abua, Ndep Antor Odu, Louis Chinweike Madubuattah, Isaac Olushola Ogunkola

**Affiliations:** a Department of Public Health, University of Calabar; b School of Basic Midwifery, Calabar; c Department of Public Health, Havilla University Nde, Ikom Cross River State; d UNESCO International Centre for Biotechnology Nsukka, Nsukka, Nigeria

**Keywords:** caregivers, neonatal sepsis, umbilical cord

## Abstract

The third leading cause of death in infants under 1-month-old is neonatal sepsis. Following the severing of the umbilical cord, bacterial infection may result in newborn sepsis and mortality. The objective of this review on cultural patterns and umbilical cord-care outcomes is to evaluate present practices and create a case for developing and implementing novel cord-care regimens in Africa.

**Materials and methods:**

In order to find published studies on cultural patterns and outcomes of umbilical cord care among caregivers in Africa from January 2015 to December 2021, we conducted a systematic literature search across six computerized bibliographic databases: Google Scholar, POPLINE, PubMed, Web of Science, Science Direct, and Scopus. As a result, a narrative synthesis of quantitative and qualitative data was employed to summarize the data from the included research.

**Results:**

There were 17 studies included in this review with 16 out of the 17 studies having a total of 5757 participants. The odds of neonatal sepsis were 13 times higher among infants whose caregivers had improper hygiene compared with those who had proper hygiene. The outcome of cord management showed that the majority (75.1%) of the umbilical cords were infected. Majority of the included studies (*n*=13) show that the respondents (caregivers) had a low level of knowledge and practice.

**Conclusion:**

This systematic review reveals that unsafe umbilical cord-care practices remained prevalent in some African regions. Home delivery is still a prevalent practice in some communities and inappropriate umbilical cord cleaning practices were common findings.

## Introduction

Highlights

- Our work revealed that unsafe umbilical cord-care practices remained prevalent in some African regions.
- Home delivery is still a prevalent practice in some communities and inappropriate umbilical cord cleaning practices were common findings.
- Our study found a discrepancy between the biomedical and community methods of cord care, these cultural methods are seen to differ across regions reflected in our study due to diverse cultural beliefs.
- The use of unhygienic materials for cord care is a common source of umbilical cord infection and neonatal death.

Neonatal mortality, or death in the first 28 days of life, accounted for 45.1% of all infant deaths worldwide in 2015, a 15% rise over the previous 15 years^1^. Infections and sepsis remained a persistent and important cause of mortality and morbidity among neonates, despite the presence of other variables^2^. The umbilical cord is a topic that comes up frequently in discussions about rites of passage. Umbilical cord-care techniques are frequently culturally regulated beliefs that serve as spiritual controls over the infant’s, mothers, and community’s well-being^3^. The cutting of the umbilical cord signifies the child’s integration into society^4,^ the child now belongs to the entire community as a result of this deed. In other words, it is a rite of departure from the child’s ancestors’ world and assimilation into the world of humans^3^.

In some African countries it is believed that in newborn care, the umbilical cord played a symbolic role. The way it was viewed and handled had far-reaching implications for the baby’s survival and well-being. The umbilical cord was a source of worry, a potential portal to illness, a test of fatherhood, and a symbol of parental duty^5^. As a result, the umbilical cord and how it was handled had a role in the baby’s current and future survival, as well as the household’s survival and well-being. In the care of the umbilical cord, people other than the mother, such as elder female relatives, played a big role^5^.

Treatment of the umbilical cord has traditionally been an important element of infant care^6^. Because of the possibility of bacterial infection of the infant’s blood supply through the broad umbilical vessel, caregivers closely monitor the umbilical cord condition before it falls off and the umbilicus is properly covered. Hygienic cord care, which entails cutting the cord with new or sterilized equipment (or a clean delivery kit) and providing correct cord care, is a frequent metric of newborn care^7^. However, different institutions and cultures manage the umbilical cord in different ways. Although some traditional health institutions advise using alcohol, others advise using antiseptics such as chlorhexidine, and yet others advise using no medication at all and simply keeping the cord clean and dry^8^.

Some of the common things applied to the chord are salty water, soot, banana ash, herbs, surgical spirit, powder, ghee, papyrus reeds, saliva, water, butter, and petroleum jelly^9^; however, they vary based on geography and cultural group^10^. Umbilical cord infections which frequently proceed to neonatal sepsis may be increased by these cultural elaborations and beliefs linked with the umbilical cord. Traditional cord-care methods that are of concern to the public’s health are frequently mentioned^10,11^. In order to reduce the high incidence of newborn sepsis in African countries, it is necessary to have a thorough grasp of the behavioural purpose that underpins conventional cord-care practices. A study of home deliveries in Tanzania found that all behaviours were acceptable for change, with the exception of cord care, where there were strong views about the need of putting something on the cord to assist it dry and come off^12^.

This systematic review addressed the following research questions; what methods do caregivers use in Tying and Cutting babies umbilical cord stumps in Africa? What Cord applications do caregivers use on babies umbilical cord stump in Africa? What is the outcome of umbilical cord care among caregivers in Africa? Despite prior systematic evidence assessments of cord-cleansing procedures, the qualitative aspect of cord-care activities has not been summarized to date. By carefully examining available evidence linked to conventional cord-care techniques and assessing the expected outcome associated with the use of traditional substances and methods and the risk of umbilical cord infection, this review addresses a gap in the literature.

## Materials and methods

This review adhered to the Preferred Reporting Items for Systematic Reviews and Meta-Analyses (PRISMA) framework.^13^. PROSPERO (#CRD42022309999) was used to register the review protocol. This systematic review is fully compliant with the PRISMA 2020 statement^13^. Checklist (see Additional File 1), Supplemental Digital Content 1, http://links.lww.com/MS9/A158. Also the study is partly compliant to the AMSTAR 2 criteria^14^. Checklist (see Additional File 2), Supplemental Digital Content 2, http://links.lww.com/MS9/A159.

### Data source and search strategy

**Figure 1:**
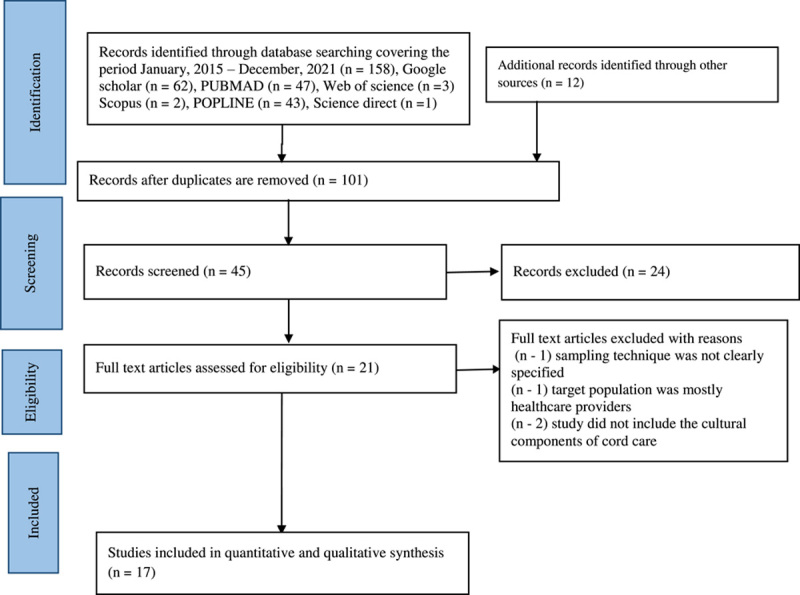
Flow chart of process of selecting articles for review.

Two members of the review team (M.A. and L.A.) conducted an independent evaluation on the cultural patterns and outcomes of umbilical cord care among African caregivers using published data from January 2015 to December 2021. The search was limited to articles that were available online, articles that mentioned cultural patterns of umbilical cord care in any part of Africa, and articles written in English. To find relevant literature, six data repositories were searched. From 2015 through 2021, they include Google Scholar, POPLINE, PubMed, Web of Science, Science Direct, and Scopus. A manual search in four relevant peer-reviewed journals pertaining to paediatrics and mother and child health issues was also done. We used the following MeSH (Medical Subject Heading) terms and keywords to search these databases: cultural patterns AND umbilical cord AND cord care AND outcome AND caregivers AND Africa. A further search included terms like “traditional practises,” “cord care practises,” and “nursing mothers.” We additionally found and incorporated relevant articles by manually examining the reference lists of previously identified papers. The PRISMA flowchart in Fig. 1 depicts the process of selecting articles.

### Eligibility criteria

#### Inclusion criteria

The following studies were chosen for inclusion in this systematic review: (1) Studies involving caregivers of neonates’ umbilical cord stumps. (2) Research including cultural patterns of umbilical cord care. (3) Research on cord and bacterial infections, as well as neonatal death. (4)Research undertaken in the Africa region/countries, as well as a general review of interest. (5) Studies done and published during a 10-year period.

#### Exclusion criteria

The following studies were excluded from this systematic review: (1) Studies in which participants are not caregivers of neonates’ umbilical cord stumps. (2) Studies in which participants’ cultural backgrounds are not taken into account. (3) Studies that fail to mention cord and bacterial infections, as well as neonatal death. (3) Research conducted outside of Africa. (4) Studies with limited technique and result information due to full-text restrictions. (5) Research conducted outside of the 10-year time frame.

#### Risk of bias (quality) assessment

The quality of the studies that satisfied the inclusion criteria was evaluated using the Downs and Black^15,16^ checklist, and each included article was evaluated and scored on a 10-item scale (see Table 1).

**Table 1:** Quality assessment of full-text articles included in review

References	Are the study’s hypotheses, goals, and objectives clearly stated?	Are the major outcomes to be measured mentioned clearly in the Introduction or Methods section?	What are the sources, procedures, and eligibility criteria for participant selection?	Is it described how the research size was determined?	Is the study’s location and sample period described?	Is a good method of measurement description provided for each variable of interest?	Are the patients’ features clearly reported in the study?	Were the statistical procedures and analyses clearly described and reported?	Are the study’s principal findings clearly described?	Is a cautious general interpretation of data provided, taking into account objectives and results from previous studies?	Overall rating
Udosen *et al.* ^17^	1	1	1	1	1	1	1	1	1	1	10
Afolaranmi *et al.* ^18^	1	1	1	1	1	1	1	1	1	1	10
Mukunya *et al.* ^32^	1	1	0	0	1	1	1	1	1	1	8
Mohamed^19^	1	1	1	0	1	1	1	1	1	1	9
Mukunya *et al.* ^5^	1	1	0	0	1	1	1	1	1	1	8
Kaoje *et al.* ^20^	1	1	1	1	1	1	1	1	1	1	10
Uwingabire *et al.* ^21^	1	1	1	0	1	1	1	1	1	1	9
Asiedu *et al.* ^22^	1	1	1	0	1	1	1	1	1	1	9
Abegunde *et al.* ^23^	1	1	1	0	1	1	1	1	1	1	9
Ango *et al.* ^24^	1	1	1	1	1	1	1	1	1	1	10
Saleh *et al.* ^25^	1	1	1	0	1	1	1	1	1	1	9
Sacks *et al.* ^26^	1	1	1	1	1	1	1	1	1	1	10
Moraa *et al.* ^27^	1	1	1	1	1	1	1	1	1	1	10
Ekuri *et al.* ^28^	1	1	1	0	1	1	1	1	1	1	9
Chizoma *et al.* ^29^	1	1	1	1	1	1	1	1	1	1	10
Ochoga *et al.* ^30^	1	1	1	0	1	1	1	1	1	1	9
Osuchukwu *et al.* ^31^	1	1	1	1	1	1	1	1	1	1	10

The primary questions developed from the checklist used to assess the quality of the studies were as follows:

1. Are the study’s hypotheses, goals, and objectives clearly stated?
2. Are the major outcomes to be measured mentioned clearly in the Introduction or Methods section?
3. What are the sources, procedures, and eligibility criteria for participant selection?
4. Is it described how the research size was determined?
5. Is the study’s location and sample period described?
6. Is a good method of measurement description provided for each variable of interest?
7. Are the patients’ features clearly reported in the study?
8. Were the statistical procedures and analyses clearly described and reported?
9. Are the study’s principal findings clearly described?
10. Is a cautious general interpretation of data provided, taking into account objectives and results from previous studies?

The quality of each study was determined by screening and analyzing the findings in connection to current practise or recommendations or relevant research-based literature, as well as if the findings may be applied to other populations.

#### Data extraction

Two trained reviewers extracted data from the included studies independently, while another trained reviewer reviewed the retrieved data. Data were discordantly evaluated by conversation to establish consensus. The following data were gathered from each eligible study: Authors; year of publication; country of study; type of study; study design; age of participants; mean age; hypotheses; *P* value; sample size; substances and materials used in tying and cleaning the cord; outcome of the result.

#### Data analysis and synthesis

A comprehensive review of the included studies found that they were unsuitable for meta-analysis due to statistical heterogeneity caused by differences in study designs and measures^17,18^. As a result, in synthesizing results from included research, a narrative synthesis of quantitative and qualitative data was used^19^. To answer the review question, the study features and conclusions from included studies were synthesized and contextually presented using the narrative synthesis approach. The findings are provided as textual narratives with tables emphasizing key findings. The primary outcome measurements analyzed in this systematic review are (a) Methods of Tying and Cutting baby’s umbilical cord stump used by caregivers in Africa. (b) The kind of cord applications caregivers use on babies umbilical cord stump in Africa. (c) Outcome of umbilical cord care among caregivers in Africa.

## Result

### Study characteristics

Table 3 shows the summary features of all considered primary studies. There were 17 studies included for this review with 16 out of the 17 studies having a total of 5757 participants. The 17 studies are a mix of qualitative and quantitative studies. One of the studies included does not have an exact sample size^32^. The study with the least sample size had 30 as the sample size^19^. The study with the largest sample size has 817 participants^24,25^. The average sample size for the various qualifying studies included in the review is around 360 people. Participants range in age from 13 to 50 years old. According to certain studies, the average age was between 25.7 and 27.6 years^17,18,21^. The most common study design in the included studies (*n*=10) is a cross-sectional study design. A total of eight studies tested a hypothesis (Table 2).

**Table 2:** Characteristics of studies that reported the cultural patterns and outcome of umbilical cord care among caregivers in Africa and were included in the systematic review

References	Year	Study country	Type of study	Research design	Age group	Mean age	Hypotheses	*P*	Sample size	Total quality of assessment score
Udosen *et al.* ^17^	2019	Nigeria	Quantitative study	Cross-sectional design	15–49 years	29.7	Yes	0.05	388	10
Afolaranmi *et al.* ^18^	2018	Nigeria	Quantitative study	Cross-sectional study design	≤30 years and above	27.5±6 years	Yes	≤0.05	263	10
Mukunya *et al.* ^32^	2018	Uganda	Qualitative study	Randomized control trial	—	—	No	—	30	8
Mohamed^19^	2018	Egypt	Quantitative study	Two-group pre-posttestQuasi experimental design	—	25.9±5.2 and 21.4±5.4	Yes	<0.001	400	9
Mukunya *et al.* ^5^	2020	Uganda	Qualitative study	Descriptive design and Randomized control trial	24–55	—	—			8
Kaoje *et al.* ^20^	2018	Nigeria	Quantitative study	Descriptive cross-sectional study	18–48	26.7±6.0	No	0.05	358	10
Uwingabire *et al.* ^21^	2020	Rwanda	Quantitative study	Descriptive cross-sectional study	15–50	Nil	Yes	0.05	224	9
Asiedu *et al.* ^22^	2019	Ghana	Quantitative and qualitative study	Cross-sectional descriptive study	13–44 years	Nil	Yes	0.05	210	9
Abegunde *et al.* ^2^	2017	Nigeria	Quantitative study	Descriptive cross-sectional study	15–49 years	26.9±6.9–30±7.0	Yes	0.05	817	9
Ango *et al.* ^24^	2021	Nigeria	Quantitative study	Cross-sectional study	18–58 years	27.62±5.4 years).	No	Nil	363	10
Saleh *et al.* ^25^		Nigeria	Quantitative and cross sectional	Retrospective study	15–49 years	Nil	Yes	0.05	312	9
Sacks *et al.* ^26^	2015	Zambia	Qualitative study	Cross-sectional study	18–59 years	Nil	No	Nil	75	10
Moraa *et al.* ^27^	2019	Kenya	Quantitative study	case control study design	15–49 years	16.5	Yes	0.05	306	10
Ekuri *et al.* ^28^	2021	Nigeria	quantitative study	Survey design	Nursing mother	Nil	No	Nil	748	9
Chizoma *et al.* ^29^	2020	Nigeria	Quantitative study	Cross-sectional design	15–35 years and above	Nil	Yes	0.05	414	10
Ochoga *et al.* ^30^	2020	Nigeria	Quantitative study	Cross-sectional study	15–46 years	27.88±5.36	Yes	0.05	398	9
Osuchukwu *et al.* ^31^	2018	Nigeria	Quantitative study	Cross-sectional study	15–49 years	26 (12.0)	No	0.05	451	10

The included primary studies were published between 2015 and 2021, with 2015 having the most studies (*n*=5) and 2020 having the fewest (*n*=4). Based on the length of the investigations, the time spent collecting data and releasing results was less than a year. All of the papers featured were initially written in English. The stipulated language for eligibility of studies had already been stated in the inclusion criteria for the study. The majority of the included studies (*n*=10) were conducted in Nigeria. Other countries include Uganda (*n*=2)^19,20^, Egypt^32,^ Rwanda^22,^ Ghana^23,^ Kenya^28,^ and Zambia^27^. The included studies generally looked at caregivers but more specifically, participants included in those studies are mothers^17,18,20–23,26,28–34,^ healthcare workers^19,20,27^, traditional birth attendants and fathers^19–21,27^. The study location spine through both rural and urban regions in the aforementioned countries.

### Materials used in cutting and tying the cord

The 17 included studies in the review revealed various materials that were used in cutting and tying neonates’ umbilical cord stumps. There are similarities in the materials used. The materials used in cutting the cords include scissors, razor blades, farm instruments and other unspecified materials^22,24–26,29,31,33,34^. There is high usage of scissors in cutting the cords ranging from 17% to as high as 95.5%^23–25^. About six studies documented the use of razor blades^24–26,28,29,31,34^. The use range from 7 to 80%. Pre-used cutting tools were indicated for razor blades, knives, and other unspecified instruments^24,25,28^.

The materials used in tying the cord include cord clamp, strings of clothing, tailor/sewing thread, plastic materials, hair thread, and black thread^18,22,26,29,31^. The most common choice is the thread which includes tailor thread (15.7%)^18,^ sewing thread (22.6%)^26,^ hair thread (11.6%), and black thread (15.6%)^31^. The percentage of cord clamp usage ranges from 51 to 84.4%^26,31^.

### Substances used in cleaning the cord

Out of the 17 included studies for this systematic review, 94.1% (*n*=16) assessed the substances used by the various caregivers in cleaning the cord (Table 3). Core information elicited by the studies established the use of the following substances Chlorhexidine, methylated Spirit, Digluconate, Iodine, Dettol, Vaseline, Dusting powder, Engine oil, Palm oil, Breast milk, Salt, Warm water, Herbs, Cow dung, Sand, petroleum jelly, and cooking oil (Table 3). Fifty-one percent of the 17 studies used indicated methylated spirit is used for cleaning and the usage among caregivers ranges from 2.8 to 83.5%^17–31,33,34^. Some studies (*n*=6) documented the use of Chlorhexidine ranging from 5.5 to 83.5%^17,21,24–26,28^. Dung such as lizard and cow dung was used as a substance for cleaning cords^17,21,29,33^. Forty-seven percent of the 17 included studies indicated the use of herbs and herbal mixture^17,20,27,29,31,34^. Saliva and salt breast milk and other unhygienic substances were commonly used by caregivers across the selected studies (Table 3).

**Table 3:** Summary of umbilical cord-care outcome

References	Materials used in cutting and tying the cord	Substances used in cleaning the cord	Outcome of the result
Udosen *et al.* ^17^	—	Substances used in cleaning the cord Chlorhexidine 131 (33.7%). Methylated Spirit, 204 (52.6%), Digluconate 97 (25%), Iodine 73 (18.8%), Dettol 9 (2.3%) .Vaseline 72 (18.6%), Dusting powder 14 (3.6%), Engine oil 58 (14.9%), Palm oil 88 (22.7%), Breast milk 72 (18.6%) Salt 87 (22.4%), Warm water 132 (34%), Herbs 116 (29.9%), Cow dung 37 (9.5%), Sand 143 (36.9%)/	Respondent’s level of Practice towards umbilical cord care was good.
Afolaranmi *et al.* ^18^	Materials used in tying the cord Cord clamps 273 (73.1%) Strings of clothing 36 (11.1%), Tailor’s thread 51 (15.7%).	Substances used in cleaning the cord Methylated spirit, (73.2%), Salt with saliva81 (25.0%),Hot water 203 (62.7%), Herbs 13 (4.0%).	The majority of responders were found to engage in overall good cord-care practices, as evidenced by the use of methylated spirit exclusively.
Mukunya *et al.* ^32^	—	Substances used in cleaning the cord Traditional substances.	The use of chlorhexidine on the umbilical cord stump at delivery was appropriate as a supplement rather than a replacement for customary drugs. The use of chlorhexidine on the umbilical cord stump during birth was appropriate as a supplement rather than a complete replacement of customary drugs.
Mohamed^19^	—	—	The findings found that just 0.5% of newborns in the intervention moms’ group developed umbilical cord infection symptoms, and 99.5% had their cord sloughed off by the end of the 2 weeks. The same table demonstrates that the practise was unsatisfactory in the control group, and the mean scores in the pre, immediate, post, and follow up tests (after 1 month) of the intervention programme were similar, with no statistically significant differences.
Mukunya *et al.* ^5^	—	Substances used in cleaning the cord Kyogero, a local herbal concoction, powder, banana (plantain) powder, as, soap, ordinary saline, tealeaf, ghee kiyondo (a local herb), and faeces (lizard and cow). sprit, saliva, and mushroom	The umbilical cord was found to pose an immediate threat to the child’s survival as well as long-term well-being if not handled with care.
Kaoje *et al.* ^20^	—	Substances used in cleaning the cord Chlorhexidine 91 (32.5%), Mathylated spirit114 (40.7%), Hot water 137 (49%), Toothpaste 84 (30.0%), Handkerchief/clean rag soaked in tepid water 29 (10.4%), Apply anything 105 (37.5%)	According to the findings of the study, home delivery is still a common practise in the community. Inappropriate umbilical cord washing methods were frequent, and both the mother and infant received very little postnatal care.
Uwingabire *et al.* ^21^	Materials use in cutting the cord Scissors to cut the cord 214 (95.5%) Materials used in tying the cord -Plastic material to used to tie the cord 204 (91.0%).	Substances used in cleaning the cord Vaseline ointment 51 (23.0%), and Movit -Ointment 34 (15.2%) -Air drying the cord naturally 157 (70.0%).	The study on umbilical cord care at five Rwandan health clinics found that moms had a low level of awareness and practise.
Asiedu *et al.* ^22^	—	Substances used in cleaning the cord Methylated spirit 35.7% (75) Shea butter, toothpaste, chalk, sand, salt, petroleum jelly, baby lotion, penicillin and amoxicillin. 64.3% (135)	Community cultural ideas determine how long mothers/caregivers accommodate the umbilical chord before it falls off. Mothers who used more acceptable cord dressing, on the other hand, had newborns whose umbilical cords separated from the stump after 4 days.
Abegunde *et al.* ^2^	Materials used for cutting the cord New razor blades 75% and 80% Scissors 17% and 23% pre-used razor blades, knives, and other unspecified instruments 7% and 9%	Substances used in cleaning the cord Chlorhexidine, ash, methylated spirit, hot compress, local herbs, and cow dung were the most commonly reported cord-care products topically applied to dress freshly cut umbilical stumps Hot compress and local herbs were used in cord dressing in the majority of the deliveries.	Unsafe umbilical cord-care practises persisted in Nigeria’s Bauchi and Sokoto states, despite the recent introduction of chlorhexidine digluconate 7.1% gel, which shifted cord-care behaviours toward safer methods among public health workers. TBAs, friends, and relatives played the most important early postpartum roles, and they typically continued to employ risky cord-care procedures such ash, cow dung, and hot compresses.
Ango *et al.* ^24^	Materials used in cutting the cord New razor blade (46.8%) Surgical blade (40.8%) Materials used in tying the cord Cord clamp (51.0%) sewing thread 22.6%, Hair thread 11.6%, String of cloth6.9%, and Bandage 6.9%	Substances used in cleaning the cord Methylated spirit/chlorhexidine (83.5%)	Respondents in this survey indicated appropriate knowledge; however, respondents’ practise of umbilical cord care was unsatisfactory.
Saleh *et al.* ^25^	—	Substances used in cleaning the cord Charcoal or cow dung	The findings show that in the six provinces, women who did not treat the umbilical cord of their newborn babies in a sanitary manner are 68% (147/216) (*n*=147) and those who did treat the umbilical cord of their newborn babies in a sanitary manner are 32% (69/216) (*n*=69). Overall, there was a considerable difference in the proportions of people obtaining sanitary cord care according on province (range: 17–58%).
Sacks *et al.* ^26^	Materials used in cutting the cord Old or new razor blades were used for cuttings	Substances used in cleaning the cord Petroleum jelly, commercial baby lotion, cooking oil breast milk. Powders made of roots, burnt gourds or ash black powder	A common documented practise of applying hazardous substances to the skin and umbilical cord may increase exposure to invasive infections. Mothers applied various substances to their children’s skin and umbilical cords, most often powders made of burnt roots or ash, with extra precautions for premature babies.
Moraa *et al.* ^27^	—	Substances used in cleaning the cord The majority of moms (64%, *n*=197) reported using chlorhexidine/surgical spirit. A little more than a third of the cases (35.6%, *n*=37) had surgical spirit/chlorhexidine administered, compared with around four-fifths (79.2%, *n*=160) of the controls. Concerningly, saliva/ash was used in 10.6% (*n*=11) of the cases compared to 2.5% (*n*=5) of the controls.	The proportion of mothers with inadequate hygiene was 35.3%: 72.1% among cases and 16.3% among control caregivers. The risks of neonatal sepsis were 13 times higher in babies whose caregivers were clean (OR=13.24; 95% CI: [7.5; 23.4]). None of the neonatal or maternal variables confounded the association between umbilical cord cleanliness and infant sepsis. This odds ratio’s PAF was 66.7% (95% CI: 62.5; 69.03).
Ekuri *et al.* ^28^	Materials used in cutting the cord Razor blade Materials used in tying the cord Hair thread	Substances used in cleaning the cord Herbal leaves Cow dung Application of hot compress Breast milk	This study found that traditional beliefs had an impact on the neonatal umbilical cord. According to the findings of this study, 43.8% of respondents agreed that in their community, some herbal leaves are put on the umbilical stump so that it falls off in a few days.
Chizoma *et al.* ^29^	Materials used in tying the cord Proper material used to tie the cord 121 (29.2%)	Substances used in cleaning the cord Proper cleaning agent used 396 (95.7) Mentholatum balm	The mean practice score of umbilical cord-care practices is 12.3±1.2. 254 (61.4%) respondents practiced good cord care
Ochoga *et al.* ^30^	Materials used in cutting the cord Scissor 159 (39.9%) Razor blade 109 (27.4%) Materials used in tying the cord Cord clamp 336 (84.4%) Black thread 62 (15.6%).	Substances used in cleaning the cord Chlorhexidine 22 (5.5%) Methylated spirit alone 272 (68.3%) Methylated spirit and another substance11 (2.8%) . Vaseline 58 (14.6%). Oil/Vaseline 28 (7.0%), Toothpaste 21 (5.3%). Herb 14 (3.5)	Mothers frequently use methylated spirit for cord care. The WHO-recommended level of chlorhexidine is relatively low, necessitating more active efforts to educate women about the use of chlorhexidine for cord care.
Osuchukwu *et al.* ^31^	Materials used in cutting the cord knife 187 (41.6) razor blade 165 (36.7) sterile scissors/surgical 94 (20.9) farm instrument 4 (0.8)	Substances used in cleaning the cord Methylated spirit 224 (49.8%) Dettol 88 (19.6%) Saliva and salt 44 (9.8%) Herbal preparations 44 (9.8%). Toothpaste 16 (3.6%) Hot water 4 (0.9) Other unhygienic substances at base of stump314 (69.8%)	The results of cord management revealed that 338 (75.1%) of the umbilical cords were contaminated.

OR, odds ratio.

### The outcome of umbilical cord-care practices among caregivers

The findings of the 17 included research are presented in terms of similarities and differences (Table 3). Only 23.5% (*n*=4) of the 17 included studies for this systematic review reveal an outcome of a good or appropriate level of cord-care practice among respondents^17,26,28,30^. Some studies^19,23,29,32^ identified cultural and traditional behaviours that influence care cord among participants. One study found that traditional beliefs had an effect on the neonatal umbilical cord^19,29^. According to one study, 43.8% of respondents agreed that in their community, some herbs and leaves are applied to the umbilical stump^29,^ and that the use of chlorohexidine on the umbilical cord stump at birth was acceptable as an addition rather than a total replacement of traditional substances^19^.

Mohammed reported that just 0.5% of the neonates in their intervention moms’ group showed umbilical cord infection symptoms, and 99.5% of their cords sloughed off before the end of the 2 weeks^29^. Mothers applied various substances to the skin and umbilical cord, most often powders composed of burnt roots or ash, which may increase exposure to invasive infections, according to Sacks *et al.* ^27^. The odds of neonatal sepsis were 13 times higher among infants whose caregivers had improper hygiene compared to those who had proper hygiene^28^. The outcome of cord management showed that the majority (75.1%) of the umbilical cords were infected^34^. Majority of the included studies (*n*=13) shows that the respondents (caregivers) had a low level of knowledge and practice^19–23,27–34^.

Furthermore, unsafe umbilical cord-care practices remained prevalent in some regions^21^. Home delivery is still a prevalent practice in some communities and inappropriate umbilical cord cleaning practices were common findings^19^.

## Discussion

This systematic review investigated the cultural patterns and outcomes of umbilical cord care among caregivers in Africa, encompassing various materials used for cutting and tying neonates’ umbilical cord stumps in 17 studies from across the continent. Owing to the statistical heterogeneity stemming from the diverse study designs and measurements, a narrative synthesis of quantitative and qualitative data was employed to synthesize the findings from the included studies.

The study incorporated a mix of qualitative and quantitative research designs. Our findings revealed similarities in locally sourced cutting materials for cord care, which deviate substantially from standard healthcare practices. This is consistent with research from low- and middle-income countries such as India, Bangladesh, and Sri Lanka^35,36^, where caregivers used similar unsanitary objects like knives, razor blades, scissors, and farm implements^28^. Although this study emphasizes traditional cord-care practices, the motivation to care for an infant’s umbilical cord is universally present across cultures to promote healing, prevent infection, and accelerate cord separation, as demonstrated by research in Ethiopia, Ghana, Uganda, Zambia, and Senegal^37,38^.

The perceived fear of diseases like tetanus commonly influenced the cultural promotion of using a new blade to cut the cord to prevent neonatal tetanus^38^. Appropriate umbilical care is essential for maintaining a sanitary cord and can be achieved through methods such as applying methylated spirit/chlorhexidine to the cord’s base, air drying the cord to enable spontaneous healing, or sponge-bathing neonates without submerging them in water^28^. We identified substances used in cord cleaning that increased the risk of infection and mortality in neonates, contradicting the WHO recommendation of dry cord care or the use of chlorhexidine in low mortality settings (less than 30 deaths per 1000 births)^28,39^. Only six studies reported chlorhexidine use in cord washing and disinfection, indicating inadequate antiseptic usage and neglect of its potential to prevent cord sepsis by applying chlorhexidine gel to the umbilical cord stump after cutting and during the first 7 days^28,39^.

The umbilical cord is known to produce secretions which makes mothers uncomfortable. A variety of unsanitary things were recognized as being utilized in cord cleaning; substances such as cow dung, sand, herbs, and dusting powder were seen to absorb these secretions and hasten the drying of the umbilical cord^6,40^. According to prior research, the usage of such dirty things is a plausible source of infection because they are likely infected with bacteria/spores and are significantly associated with an elevated risk of omphalitis and newborn sepsis^28^.

Moreover, our study found a discrepancy between the biomedical and community methods of cord care, these cultural methods are seen to differ across regions reflected in our study due to diverse cultural beliefs. Some of the beliefs surrounding the dropping of the neonatal cord are perceived to be the time when the baby is believed to become human or less vulnerable to illness or to people with bad intentions, bringing to light the importance of culture as a driver of practice of cord care and its underestimated subtle contributions to neonatal morbidity and mortality particularly in resource poor countries^38^. Practice of appropriate cord care among caregivers were significantly poor in our study representing over 70% of our findings as was similar in southern Nepal where there was low coverage of clean cord care^41^. Whereas, a study in Ethiopia showed good knowledge and practice of cord care and the reasons for these differences may range from the cultural background of the mothers to access to health facility and health professionals. Nevertheless, the use of methylated spirit as a cleaning substance was consistent among caregivers across selected studies with 51% indicating its use to keep the stump clean, dry, and promoting quick cord separation^18^. Some of the substances applied to the stump are believed to heal internal sores, prevent environmental dangers and illness as opposed to a contrary study in India where nothing is being applied to the cord, not to practice dry cord care but because of low awareness^41^.

Amid high rate of unhygienic substances and the refusal of some caregivers to adopt orthodox healthcare approach, our results revealed high rates of infected cords and neonatal sepsis among infants as an outcome of poor cord care which are consistent with cultural beliefs and practices. Similar studies have discussed situations of how umbilical infections represent themselves in infants and they include; black bumps in the cord, distended abdomen^40,^ and cord with redness, discharge or pus^42^. Conversely, if good cord hygiene was observed, cases of umbilical cord infections, omphalitis and neonatal sepsis would have been averted, expressing the need for knowledge advancement of neonatal care and proper hygienic practice of cord care.

### Implications of the study

Thorough literature review shows that this systematic review on cultural patterns and outcome of umbilical cord care among caregivers is the first in Africa and among the target group. The review focused on materials, substances used and outcome of management. From the results, the knowledge of umbilical care and practice was low, unsafe umbilical cord-care practices remained prevalent in some of the African communities compared with developed countries such as the United States. The study provides a basis for comparison between cultural practices regarding cord care and other medical methods such as use of methylated spirit and chlorhexidine gel. Majority of studies revealed that herbs, leaves, cow dung, sand, and other unhygienic substances were applied to the umbilical cord, this explains the unfavourable outcome of cord care. It is therefore recommended that further studies on knowledge and practice of caregivers including nursing mothers and healthcare providers on umbilical cord care be carried out especially in low-income and middle-income countries.

### Strengths and limitations of the review

This review had some limitations as well as strengths. The review focused only on studies written in English language, this can create a kind of biases for studies that were not presented in English language. Also, the study considered only the cultural components of umbilical cord care whereas there can be other factors that influence umbilical cord care. In the same vein, the study was limited to only caregivers without considering the important role of the healthcare workers in child care. However, the study also had strengths, the review had both the quantitative and qualitative part, it also utilized studies with different designs, and this provided more insight into the study, participants’views and experiences were explored. Studies that did not meet the inclusion criteria were excluded such as studies without clear explanation of the sampling procedure and studies conducted outside Africa. Heterogeneity was checked hence a narrative synthesis of quantitative and qualitative data was adopted in synthesizing results from included studies.

## Conclusions

Summarily, this systematic review revealed that unsafe umbilical cord-care practices remained prevalent in some African regions. Home delivery is still a prevalent practice in some communities and inappropriate umbilical cord cleaning practices were common findings. The use of unhygienic materials for cord care is a common source of umbilical cord infection and neonatal death. These unhealthy cord-care practices were observed across several studies in this review. There is therefore the need to assess the knowledge of caregivers and other significant others have regarding cord care. Also, further studies need to be carried out to evaluate the effectiveness of some of these local substances used on the cord. Government in collaboration with healthcare providers should setup rigorous health education/promotion activities in partnership with local radio stations, adopting the social media options and the use of local language support, to educate women and caregivers about the effect of unsafe umbilical cord practices.

## Ethical approval

NA.

## Consent

Not applicable.

## Source of funding

This research received no external funding.

## Author contribution

Conceptualization: M.A.A., A.N.O., L.M.C. Data curation: M.A.A., L.M.C. Formal analysis: I.O.O. Investigation: M.A.A., A.N.O., L.M.C. Methodology: M.A.A., A.N.O., L.M.C. Project administration: M.A.A., A.N.O., L.M.C. Resources: M.A.A., A.N.O. Supervision: A.N.O. Validation: A.N.O. Writing—original draft: M.A.A., A.N.O., L.M.C. Writing—review and editing: M.A.A., A.N.O., I.O.O.

## Conflicts of interest disclosure

The authors declared no conflicts of interest.

## Research registration unique identifying number (UIN)

1. Name of the registry: Not applicable.
2. Unique Identifying number or registration ID: Not applicable.
3. Hyperlink to your specific registration (must be publicly accessible and will be checked): Not applicable.

## Guarantor

Isaac Olushola Ogunkola.

## Provenance and peer review

Not commissioned, externally peer-reviewed.

## Supplementary Material
